# Stromal Cells from Human Decidua Exert a Strong Inhibitory Effect on NK Cell Function and Dendritic Cell Differentiation

**DOI:** 10.1371/journal.pone.0089006

**Published:** 2014-02-20

**Authors:** Daniele Croxatto, Paola Vacca, Francesca Canegallo, Romana Conte, Pier Luigi Venturini, Lorenzo Moretta, Maria Cristina Mingari

**Affiliations:** 1 Department of Experimental Medicine, University of Genoa, Genoa, Italy; 2 Giannina Gaslini Institute, Genoa, Italy; 3 IRCCS AOU San Martino-IST (National Institute for Cancer Research), Genoa, Italy; 4 DINOGMI, University of Genoa, Genoa, Italy; University of Florence, Italy

## Abstract

Stromal cells (SC) are an important component of decidual tissues where they are in strict proximity with both NK and CD14^+^ myelomonocytic cells that play a role in the maintenance of pregnancy. In this study we analyzed whether decidual SC (DSC) could exert a regulatory role on NK and CD14^+^ cells that migrate from peripheral blood (PB) to decidua during pregnancy. We show that DSCs inhibit the IL15-mediated up-regulation of major activating NK receptors in PB-derived NK cells. In addition, the IL15-induced NK cell proliferation, cytolytic activity and IFN-γ production were severely impaired. DSCs sharply inhibited dendritic cells differentiation and their ability to induce allogeneic T cell proliferation. Indoleamine 2,3-dioxygenase (IDO) and prostaglandin E2 (PGE2) mediated the inhibitory effect of DSCs. Our results strongly suggest an important role of DSCs in preventing potentially dangerous immune response, thus contributing to maintenance of pregnancy.

## Introduction

Natural killer (NK) cells are major effectors of the innate immunity and are generally thought to play a fundamental role in antiviral and antitumor responses [1], [2]. Although the prevalent role of NK cells is to defend the host against infections and, possibly, tumors, recent studies have indicated that they may also display additional functional capabilities [3]–[5]. Human NK cells function is regulated by both inhibitory i.e. Killer Ig-like receptors (KIRs) and CD94/NKG2A and activating receptors including NKp46, NKp30 and NKp44 termed Natural Cytotoxicity Receptors (NCR), NKG2D, DNAM-1 and CD16 [6]–[9].

In human pregnancy the balance between active immunity and tolerance at the site of contact between mother and fetus, i.e. the decidua, is of critical importance. Thus, while effective immunity must be maintained to protect the mother from harmful pathogens, tolerance should be induced towards fetal antigens. Indeed, since the fetus represents a semi-allograft, during pregnancy mechanisms should exist to prevent allograft rejection [4], [10], [11]. During the first trimester of pregnancy NK cells represent 50–70% of the total lymphoid cells present in the decidual tissue and display a unique functional profile [10]–[15]. Decidual NK (dNK) cells are CD56^bright^, CD16^−^, KIR^+^ and display normal levels of activating NK receptors [10], [16]. Although they contain high amounts of cytolytic granules, they are poorly cytolytic [10], [14], [17], [18]. Upon interaction with trophoblast cells, dNK cells release high amounts of cytokines/chemokines that play a major role in tissue remodeling and/or neo-angiogenesis [4], [11], [13]–[15], [19]–[21].

Little information exists on the origin of dNK cells. They could derive from peripheral NK cells recruited in decidua at early stages of pregnancy or originate *in situ* from precursors [22]–[25]. In this context, previous studies described the presence of CD34^+^ cells in decidual tissues and of immature NK cells in endometrial tissue; both cell populations were capable of differentiating into mature dNK cells *in vitro* [22], [23], [25], [26]. Remarkably mature dNK cells could also be obtained upon co-culture with decidual stromal cells (DSC) in absence of added cytokines, underscoring the role of these cells in inducing NK cells differentiation in decidual tissues [22], [26].

Another abundant cell population (∼20%) in decidual tissues during the first trimester is represented by CD14^+^ myeloid cells (referred to as dCD14^+^ cells) [27]–[29]. These cells show phenotypic characteristics intermediate between polarized macrophages (M2) and dendritic cells (DCs) [27]–[30]. Histochemical analysis revealed that dNK cells could reside in close association with dCD14^+^ cells, a condition that may favor functional interaction between the two cell types [27]. Indeed, upon *in vitro* co-culture with dNK cells, dCD14^+^ cells acquired the capability of inducing CD4^+^ regulatory T cells (Tregs) [31]. In turn, Tregs are thought to play a major role in the inhibition of maternal immune responses and in tolerance induction [32]–[34].

Another important cellular component in decidual tissues is represented by stromal cells (DSCs). SCs are non-hematopoietic cells exhibiting multilineage differentiation capacity [35], [36] and the capability of mediating immunomodulating and anti-inflammatory effects and may include different cell subsets (i.e. fibroblasts and mesenchimal stem cells) [35], [37]–[43]. In particular, SC can be easily isolated from various tissues: i.e. Bone marrow (BM), Umbilical cord blood (UCB), fat, gingiva, placenta and other tissues [35], [37], [40], [43].

Previous studies revealed that SCs could modulate different functional capabilities of T, B, NK, monocyte/macrophages, DC and neutrophils while inducing Tregs [39], [42], [44]–[49]. The mechanisms by which SCs exert their inhibitory effect involve both cell contact [47], [48] and soluble factors, including Indoleamine 2,3-dioxygenase (IDO)–induced L-kynurenine, transforming growth factor beta (TGF-β), interleukin (IL)10, prostaglandin E2 (PGE2), nitric oxide (NO), and certain chemokines or cytokines [26], [40], [41], [45], [46]. Previous studies indicated that DSC were involved in the induction of feto-maternal tolerance during pregnancy [34], [39], [40], [50]. However, the role of DSC in the immuno-modulation of NK cells and myeloid cells, migrating to decidua from PB when pregnancy is established, is not fully clarified. In this study we analyzed the effect of DSC on PB-NK cell function and on CD14^+^ cells undergoing *in vitro* differentiation towards DCs. We show that DSCs can exert a potent inhibitory effect on NK cell proliferation, cytotoxicity and IFN-γ production. Perhaps more importantly, they can block DC differentiation, thus preventing fetal antigen presentation. The DSC-induced inhibition is primarily mediate by IDO and PGE2. Taken together these results strongly suggest an important role of DSC in the induction of tolerance towards the fetal allograft.

## Materials and Methods

### Isolation and Culture of Cell Populations

Lymphocytes were isolated from PB from healthy donors using Ficoll-Hypaque density gradient either directly or after enrichment for NK cells using RosetteSep (StemCell Technologies, Vancouver, British Columbia, Canada). After centrifugation, the recovered NK cells were assessed for purity by flow cytometry. Only those populations displaying more than 97% of CD56^+^CD3^–^HLA-DR^–^CD14^–^ NK cells were used. CD14^+^ cells were positively selected from PBMCs of healthy donors, using the MACS CD14 MicroBeads (Monocyte Isolation Kit, Miltenyi Biotec) according to the manufacturer’s instructions. Purity of separated monocytes was assessed by flow cytometry and was more than 98% of CD14^+^ cells.

We obtained decidua samples at 9–12 weeks of gestation from single pregnancies of mothers requesting termination of the pregnancy for social reasons. Decidual tissues from different subjects, after being carefully freed from trophoblast, were washed in PBS and cells were isolated with GentleMacs, Miltenyi Biotec and analyzed as previously described [17]. The relevant institutional review boards approved the study and all patients gave their written informed consent according to the Declaration of Helsinki. Ethical committee AZIENDA OSPEDALIERA UNIVERSITARIA “San Martino”, Genova - Italy, U.O. Affari generali e Legali, Delibera n° 0625 del 29-07-2009. DSCs were isolated according to the methods described in previous studies [39], [50]. Cell suspensions were plated and cultured in the presence of 10% fetal calf serum (FSC)-supplemented RPMI medium 1640 (BioWhittaker, Lonza). After 48 hours, we removed non-adherent cells to separate the adherent cell fraction. Half the medium volume was replaced twice a week. DSCs were used in the experiments only after 1 to 2 expansion passages to ensure depletion of monocytes/macrophages. When the cultures nearly reached confluence, harvested by treatment with EDTA 2% solution and replated at 5×10^5^ cells per 75-cm^2^ tissue-culture flasks. DSCs were confirmed to be vimentin positive (>98%) and cytokeratin negative by flow cytometry, which is in line with published studies [39], [50], [51]. In order to analyze the staminal properties, DSC were cultured with adipocyte supplement medium (Miltenyi) and analyzed with RedOil staining (Sigma-Aldrich).

### mAbs and FACS Staining

Cells were pre-incubated with human IgG (Baxter). We purchased the following specific mAbs from Miltenyi Biotec: CD3 (IgG2a), CD14 (IgG2a), CD56 (IgG1), CD314 (NKG2D) (IgG1) and CD107a (IgG1); from BD Biosciences IFN-γ (IgG1), CD73 (IgG1), Granzyme A (IgG1), CD44 (IgG2b) and RANK (IgG1); from Beckman Coulter-Immunotech CD337 (NKp30) (IgG1), CD335 (NKp46) (IgG1) and CD336 (NKp44) (IgG1); from R&D Systems ULBP1 (IgG2a), ULBP2 (IgG2a), ULPB3 (IgG2a) anti-IL8 (IgG1), IL15α (IgG2b), CD122 (IgG1) and CD132 (IgG2a); from Ancell CD29 (IgG1), CD105 (IgG1), CD106 (IgG1) and perforin (IgG2b); from Amgen ULBP-4 (IgG1); from BioLegend CD1a (IgG1), CD90 (IgG1) and RANKL (IgG2b); from Life Technologies Granzyme B (IgG1); from Santa Cruz biotechnology Vimentin (IgG1); from LifeSpan BioSciences Cytokeratin-7 (IgG1); from Abcam Desmin (IgG). CD146 (IgG2a), M7E22 mAb anti-CD54 (IgG1), D1–12 mAb anti-HLA-DR, W6.32 mAb anti-HLA-I (IgG2a), L95 mAb anti-PVR (IgG2a), L14 mAb anti-Nectin-2 (IgG2a) and BAM195 mAb anti-MICA (IgG1) were kindly provided by Pende D. (Genoa, Italy). We purchased secondary conjugated-specific mAbs from Invitrogen or Southern Biotech. All samples were analyzed on Gallios Flow Cytometer (IL-Beckman Coulter). Data analysis was done using FlowJo software.

### NK Cell/DSCs Co-culture

NK cells were cultured in RPMI-1640 10% FCS and 10 ng/mL IL15 (Miltenyi Biotec), in 24-well flat bottom plates either in the absence or in the presence of DSC (NK/DSC ratio: 5/1). At the indicated time intervals NK cells were harvested and analyzed. When indicated, 0,25 mM 1-MT (Sigma–Aldrich) and/or 5 uM NS-398 (Cayman Chemicals) and 10 µg/ml anti-Jagged-1 (R&D systems) were added at the onset of co-cultures. In some experiments, transwell inserts (Corning incorporated, 6,5 mm/0,4 µm) were used to separate NK from DSCs.

### Cytolytic Activity and Proliferation Assay

We analyzed cytotoxicity of NK cells cultured alone or with DSC in a 4 hours ^51^Cr–release assay against different target cell lines (K562 erythroleukemia and FO1 melanoma cell lines). We performed experiments in duplicates and with different Effector/Target (E/T) ratio. Data are expressed as percentage of target cell lysis. FO-1 cell line was isolated in 1991 in Dr. S. Ferrone Laboratory (New York Medical College). Several papers provided the FO-1 HLA typed by SSPO analysis (HLA-A25 and -B8). We confirmed and authenticated it in our lab by PCR-SSP HLA class I typing (HLA-A25, -B08, -BW6, and -CW7) [52], [53]. HLA Class I-negative K562 (human erythroleukemia) cell line was donated by CRB-IST cell bank ICLC. Both the cell lines were used within 6 months of resuscitation of original cultures.

To evaluate the DSC-mediated inhibition of NK-cell proliferation and a possible involvement of IDO and PGE2 in the suppressive effect, we used 5,6-carboxyfluorescein diacetate succinimidyl ester (CFSE) dilution method (Sigma–Aldrich). CFSE-labeled NK cells were co-cultured with IL15 (10 ng/mL, Miltenyi Biotec) either alone or in the presence of DSC (NK/DSC ratio of 5∶1), in the absence or in the presence of IDO and/or PGE2 inhibitors and anti-Jagged-1 neutralizing mAb. At day 5 of culture CFSE-NK cells proliferation was analyzed by flow cytometry.

### Analysis of Degranulation and Cytokine Release

To evaluate degranulation (CD107a), IFN-γ and IL-8 production by NK cells after interaction with DSCs, co-culture experiments were performed. At day 5 of culture NK cells were isolated and incubated with K562 or FO1 target cells (E/T ratio of 1∶1) or PMA/ionomycin (Sigma-Aldrich) final concentration 25 ng/ml and 1 µg/ml respectively. Monensin-containing GolgiStop (BD Biosciences) at final concentration of 2 mM was added to these experiments. After 4 hours, cells were harvested, and surface and intracellular staining were performed. For intracellular cytokine staining, cells were incubated with anti–IFN-γ or with anti-IL-8 mAbs for 30 minutes at 4°C, then washed and re-suspended in PBS 2% FCS for cytofluorimetric analysis.

### Monocyte/DSCs Co-culture

Monocyte cells were cultured in RPMI-1640 10% FCS and 25 ng/ml GM-CSF (Miltenyi Biotec) and 20 ng/ml IL4 (Miltenyi Biotec) either in the absence or in the presence of DSC (NK/DSC ratio: 5/1). After 5 days monocyte-derived cells were harvested and analyzed by flow cytometry. When indicated, 1-MT and/or NS-398 and anti-Jagged-1 neutralizing mAb were added at the onset of co-cultures.

### T-cell Proliferation Assays

Monocyte-derived cells obtained, at day 5, from co-culture with DSC in the presence of GM-CSF and IL-4 were used as stimulators in T-cell proliferation assays. Monocyte-derived cells were irradiated (50 Gy) and plated at 2×10^4^ cells/well. CFSE-labeled T cells were used as responder. At day 7 of culture the proliferation of CFSE-labeled T cells were analyzed by flow cytometry.

### ELISA Assay

PGE2 levels were measured by competitive enzyme-linked immunosorbent assay (ELISA) technique using a commercially available ELISA kit (Cayman Chemicals).

### RT–PCR Analysis

Total RNA was extracted from DSC cells harvested, either freshly derived or following overnight incubation with supernatant (spt) of IL15-activated NK cells (10 ng/mL of IL15 by Miltenyi Biotec) or with 100 UI of IFN-γ (Miltenyi Biotec) or NK/DSC TW co-culture supernatant, using RNAeasy micro kit (Qiagen). Random hexamer primer cDNA was prepared by standard technique using Transcriptor (Roche). PCR amplifications were performed with the following primers: β-actin for 5′ ACTCCATCATGAAGTGTGACG; β-actin rev 5′ CATACTCCTGCTTGCTGATCC; IDO ORF for 5′ GACTACAAGAATGGCACACG; IDO ORF rev 5′ AATGTGCTCTTGTTGGGTTAC; and Jagged-1 for: 5′-AGT GAT TGA CAG CTG CAC AG-3′; Jagged-1 rev: 5′-ACA TGT GCC CCC ATT ATG G-3′. Amplifications were performed for 30 cycles (30 s at 95°C, 30 s at 58°C, 30 sec at 72°C) using Platinum TAQ (Invitrogen). PCR products (249 bp fragment for β-actin, 1244 bp for IDO and 559 bp for Jagged-1) were run on a 1,5% agarose gel and visualized by ethidium bromide staining. Relative quantitation of gene expression was performed in RT-PCR utilizing Platinum SYBR Green qPCR SuperMix-UDG (Invitrogen) and 18S as housekeeping gene. Amplifications were performed with Mastercycler ep Realplex (Eppendorf) in a 20 µl final volume, using primers at 300 nM for 40 cycles (15 seconds at 95°C, 30 seconds at 58°C). A dissociation curve was carried out at the end of 40 cycles to confirm specificity of amplification. The following primers were used: 18S-for 5′-CGGCTACCACATCCAAGGAA-3′, 18S-rev 5′-GCTGGAATTACCGCGGCT-3′, VEGF-for 5′-GCA GCT TGA GTT AAA CGA ACG -3′, VEGF rev 5′-GCA GCT TGG TTT CTG TAT C-3′. Relative expression of each transcript was obtained using ΔΔC_T_ method.

### Statistical Analysis

Statistical analyses were performed with GraphPad Prism 6.00. Significance was evaluated by Wilcoxon matched-pairs signed rank test and paired t-test: a p value of less than 0.05 (*), less than 0.01 (**), or less than 0.001 (***) was considered statistically significant; n.s., not statistically significant. Column bar graphs were plotted as mean and SEM.

## Results

### 1. Phenotypic Characterization of DSCs

**Figure 1:**
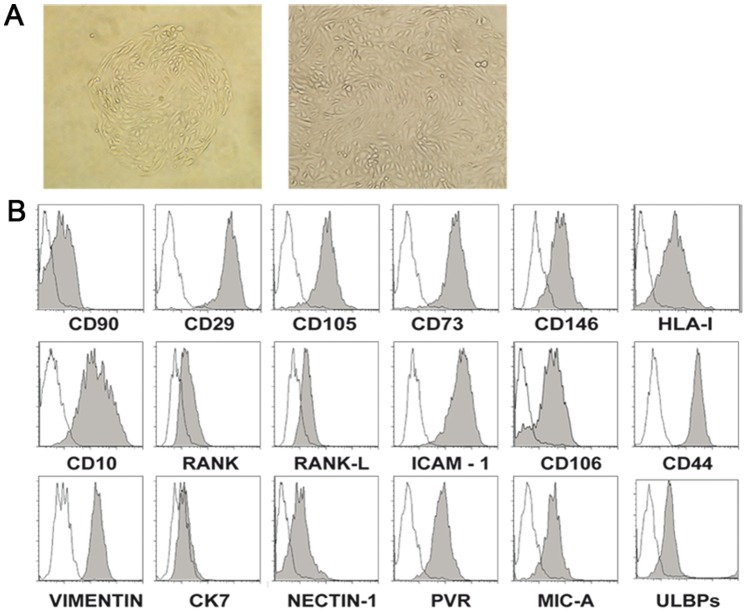
Informative surface markers expressed by DSCs. Characterization of DSCs. (A) Morphology of adherent cells isolated from decidua tissues (magnification 20X and 40X). (B) Phenotypic analysis of DSC by flow cytometry. The histograms depict the expression of different molecules (filled gray profiles) compare to negative controls (white profiles). One representative experiment out of eight performed.

After 3–4 weeks in culture, DSCs displayed a fibroblast-like morphology (Figure 1A) but showed a lower capability of proliferation (only 1–2 passages after 4 weeks of culture) than classical fibroblasts (not shown). Previous reports showed that mesenchimal stem cells exhibit staminal properties, being capable of differentiating into other cell types (including adipocytes and chondrocytes) [35], [36]. In our experimental setting using an appropriate culture medium DSCs failed to undergo differentiation towards adipocytes (not shown). Up to now, no markers specific for SCs have been identified, however, SCs of different sources may express CD105, CD106, CD10, CD90, CD73, CD44, CD146 and CD29. In addition, previous studies indicated that decidualized DSC (both *in vivo* and *in vitro*) express desmin and release prolactin [54], [55]. Therefore we analyzed the expression of these markers on DSC after 1–2 passages. As shown in Figure 1B, DSCs expressed high levels of CD10, CD29, CD105, CD73, CD146, CD54 (ICAM-1), CD106 and CD44, while classic HLA class I molecules, desmin (not shown), RANK, RANK-L and CD90 were expressed at intermediate/low levels. In addition, DSC were negative for the leukocyte markers CD45, for endothelial marker (not shown) and for cytokeratin 7 while expressed vimentin (>98% positive cells) (Figure 1B). Since we intended to study the NK/DSC interactions, DSCs were also analyzed for the expression of ligands recognized by activating NK receptors. As shown in figure 1B, the DSCs expressed Nectin-2 and PVR (ligands of DNAM-1) and ULBPs and MICA (ligands of NKG2D).

### 2. DSCs Inhibit the IL15-induced Expression of Activating Receptors, Perforins and Granzymes in PB-derived NK Cells

**Figure 2:**
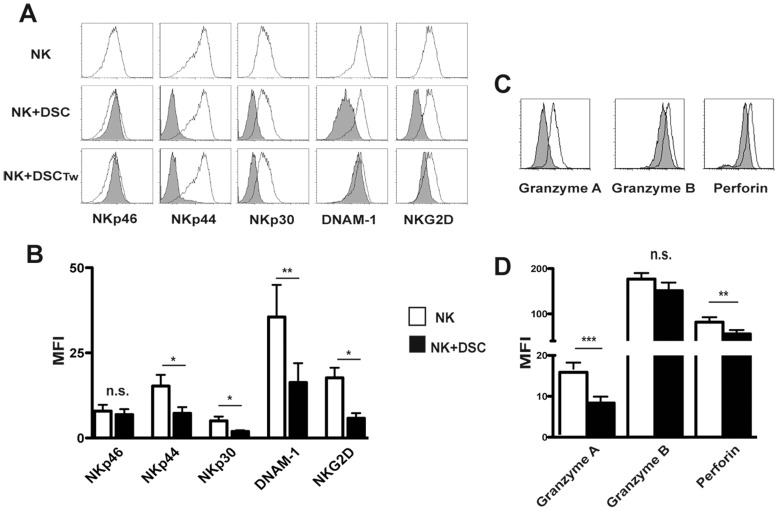
DSCs inhibit the IL15-induced expression of activating receptors, perforin and granzymes in PB-derived NK cells. (A) Expression of NKp46, NKp30, NKp44, DNAM-1 and NKG2D on IL15-activated PB NK cells, at day 5 of culture, in the absence (white profiles) or in the presence of DSCs (grey profiles) by cell-to-cell contact or by transwell (Tw) conditions. Cells were analyzed by gating on CD56^+^CD3^−^ cells. One representative experiment out of 10 performed. (B) Statistical analysis of MFI of activating NK receptors on IL15-activated PB NK cells in the absence (white bar) or in presence (black bar) of DSCs. Data is shown as a MFI±SEM of ten experiments. (C) Expression of granzyme A, granzyme B and perforin in IL15-activated PB NK cells in the absence (white profiles) or in the presence (grey profiles) of DSCs. One representative experiment out of 6 performed. (D) Statistical analysis of MFI±SEM of six experiments.

We first investigated whether DSCs could affect NK cell activation. To this end, experiments were performed either in co-cultures, ensuring cell-to-cell contact or under transwell conditions. NK cells were isolated from PB of healthy donors and cultured with IL15 (a cytokine constitutively present in decidual microenvironment) in the presence or in the absence of DSCs. After 5 days, PB-NK cells were analyzed for the expression of activating receptors and the presence of perforin and granzymes i.e. the major components of cytolytic granules. The results indicate that DSCs inhibit the IL15-induced up-regulation of NKp44, NKp30, NKG2D and DNAM-1 receptors, while the expression of NKp46 was not significantly modified (Figure 2A–B). Also under transwell conditions, DSC exerted an inhibitory effect on the expression of NKp44, NKp30 and NKG2D, while DNAM-1 was not affected (Figure 2A).

We also assessed the expression of perforin and granzymes. Intracytoplasmatic staining shows that NK cells cultured in the presence of DSC express lower levels of perforin and granzymes than NK cells cultured alone (Figure 2C–D).

DSC could mediate their inhibitory effect by influencing the expression of IL15 receptors on NK cells. To analyze this possibility, we analyzed the expression of IL15 receptors (subunits α, CD122 and, CD132) on NK cells after 24 and 48 hours of co-culture with DSC. As shown in figure S1A, the expression of IL15 receptors on NK cells was not affected.

### 3. DSCs Inhibit the NK Cell Proliferation, Cytotoxicity and Cytokine Production

**Figure 3:**
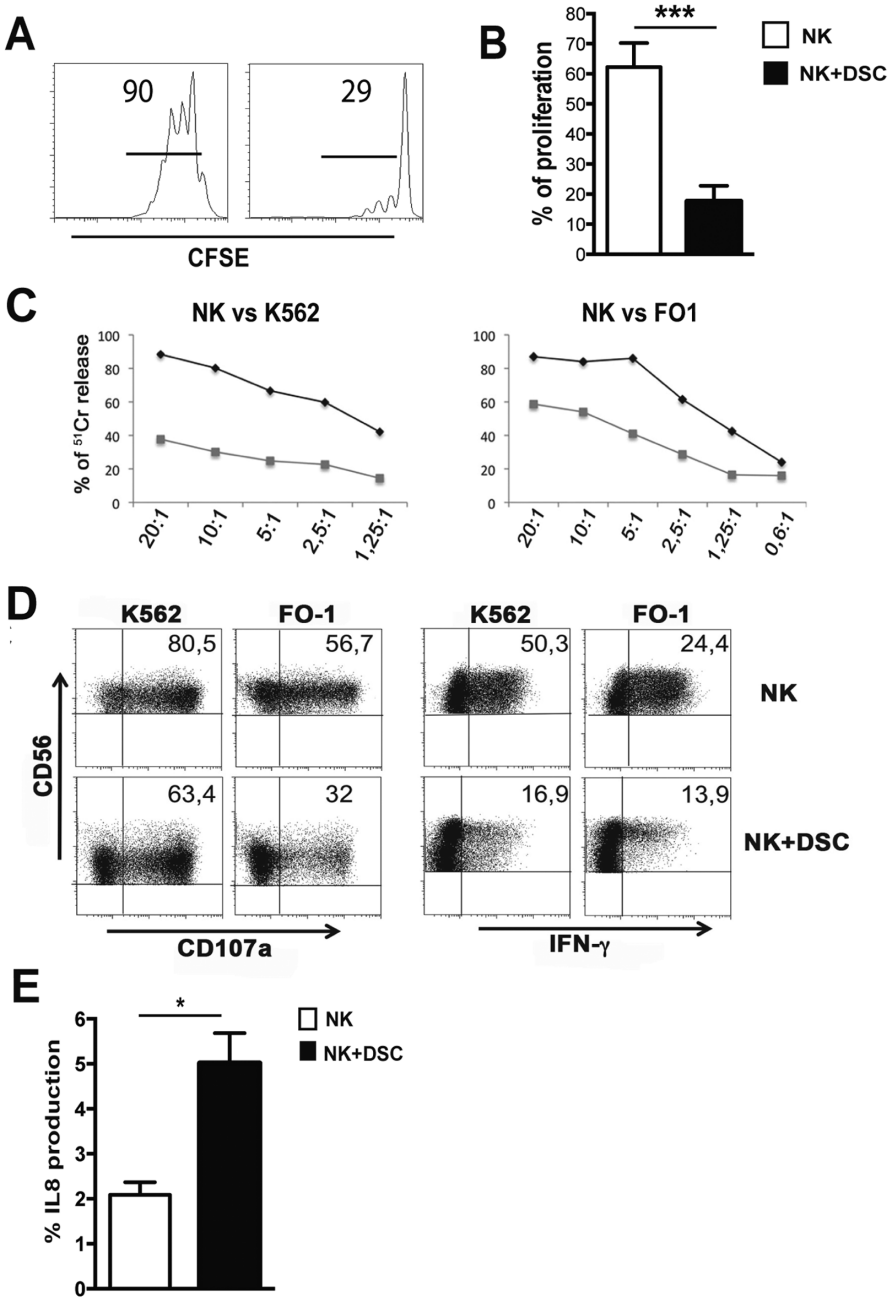
DSCs inhibit the NK cell functions. IL15-activated NK cells were cultured in the presence of in the absence of DSCs. (A) Proliferation of CFSE-labeled NK cells was evaluated at day 5 of culture by flow cytometry. One representative experiment out of 9 performed. (B) Statistical analysis of proliferating CFSE-labeled NK cells. (C) Cytolytic activity of IL15-activated NK cells in the presence (grey line) or in the absence (black line) of DSCs was evaluated by ^51^Cr release against K562 and FO1 target cell lines. One representative experiment out of 6 performed. (D) CD107a expression and IFN-γ production, after 4 h of co-culture with K562 and FO1 target cell lines by IL15-activated NK cells in the presence or in the absence of DSCs for 5 days. Cells were analyzed by gating on CD56^+^CD3^−^ cells. One representative experiment out of 7 performed. (E) Percentages of IL-8 positive cells, after 4 h of co-culture with PMA/ionomycin in IL15-activated NK cells cultured in the absence (white bars) or in the presence (black bars) of DSCs.

We next determined whether the altered expression of activating NK receptors in NK/DSC co-cultures was associated with impaired NK cell function. To this end, we evaluate the effect on NK cell proliferation, cytotoxicity and cytokine production. PB-NK cells were cultured, for 5 days with IL15 in the presence or in the absence of DSCs. Figure 3A–B, shows that DSCs sharply inhibit NK cell proliferation.

For the analysis of cytolytic activity, K562 and FO1 were used as target cells. PB-NK cells cultured alone efficiently killed both tumor cell lines. In contrast, those co-cultured with DSCs displayed a reduced cytolytic activity (Figure 3C). Analysis of PB-NK cell degranulation using the CD107a assay revealed a reduced CD107a expression in NK cells co-cultured with DSCs (Figure 3D). A similar inhibitory effect was detected on IFN-γ production (Figure 3D). Data were statistically significant (Figure S1B–C). Moreover, NK cells co-cultured with DSC acquired the capability of producing IL8, upon stimulation with K562 cells or PMA/Ionomycin (Figure 3E and S1D), and up-regulated the VEGF mRNA expression (Figure S1E). Taken together these data indicate that DSCs may influence different functional capabilities of IL15-activated NK cells.

### 4. DSCs Inhibit DC Differentiation from Peripheral Blood Monocytes

**Figure 4:**
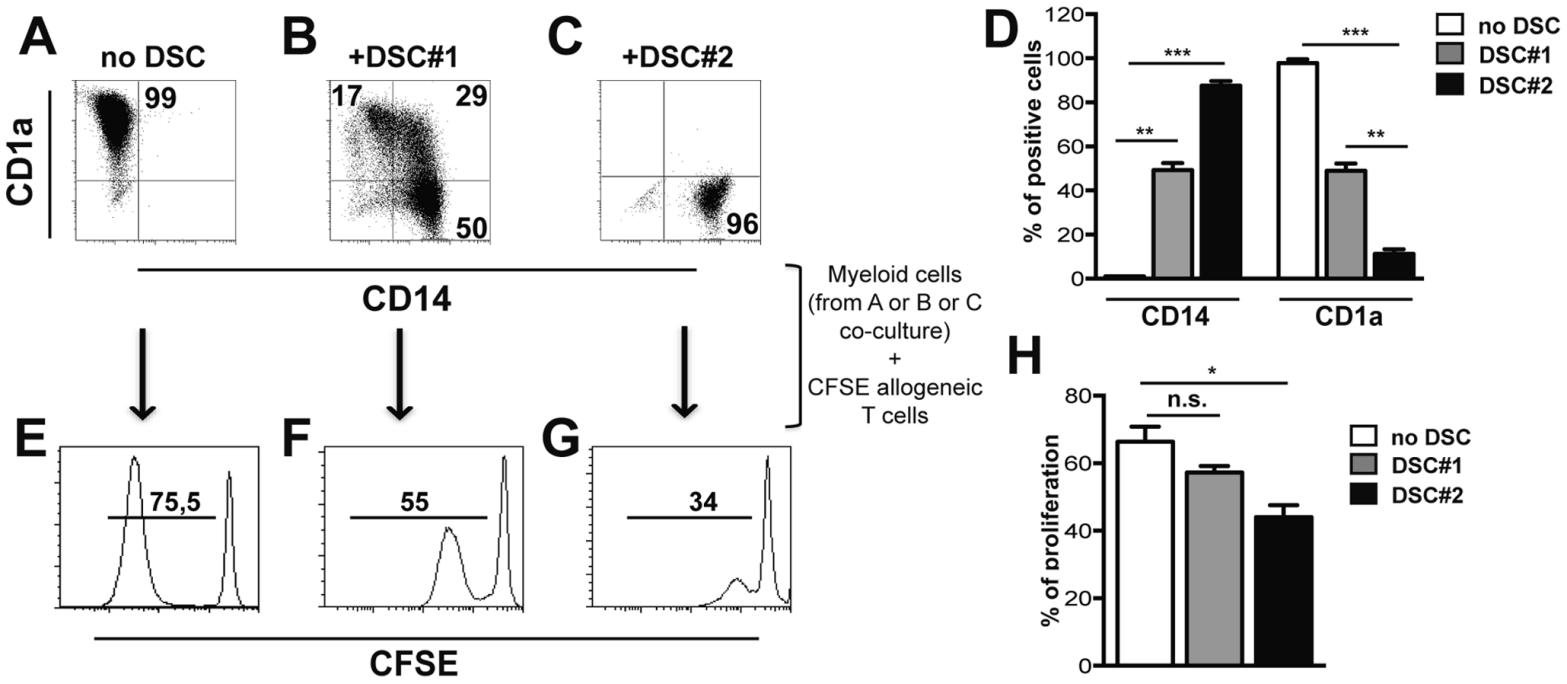
DSCs inhibit DC differentiation from peripheral blood monocytes. PB-CD14^+^ cells were cultured with GM-CSF and IL4 for 5 days in the presence or in the absence of DSCs. (A-C) Phenotypic analysis of CD14 and CD1a markers. One representative experiment out of 35 performed. (D) Statistical analysis of CD14 and CD1a expression ± SEM. (E-G) Myeloid cells were purified at day 5 from co-culture with DSCs and used as stimulator for allogeneic CFSE-labeled T cells. Proliferation of allogeneic CFSE-labeled T cells was analyzed at day 7. One representative experiment out of 8 performed. (H) Statistical analysis of proliferating CFSE-labeled T cells ± SEM.

Decidual CD14^+^ myelomonocytic (dCD14^+^) cells represent another cell population present in decidual tissues that appear to plays an important functional role during pregnancy. Notably, these cells display phenotypic features intermediate between macrophages and DC. It is possible that they are the result of interference with the process of differentiation from monocytes to DC. To investigate whether DSC could be responsible of such interference, purified PB-CD14^+^ cells were cultured with GM-CSF and IL4 in the presence or in the absence of DSCs. After 5 days of culture, the expression of CD14 and CD1a was analyzed. In the absence of DSCs, all cells lost CD14 and acquired CD1a expression (Figure 4A and D). In the presence of DSCs, two different effects were observed. Ten different preparations of DSCs (referred to as DSC#1, obtained from 10 different patients) out of 30, displayed a partial inhibition of DC differentiation. As shown in figure 4B, in the presence of these DSCs a fraction of PB monocytes underwent DC differentiation acquiring the surface expression of CD1a (Figure 4B and D). However, in the remaining 20 cases analyzed, DSCs (referred to as DSC#2, obtained from 20 different patients) strongly inhibited monocyte differentiation as revealed by the persistence of high-proportions of CD14^+^ CD1a^−^ cells (Figure 4C and D). These data suggest that monocytes migrating from periphery to decidual tissues may fail to differentiate into DC as a consequence of the interaction with DSC.

### 5. CD14^+^ Cells Co-cultured with DSCs are Poor T Cell Stimulators

Monocyte-derived cells, cultured either in the presence or in the absence of DSCs for 5 days, where analyzed for their ability to induce allogeneic T cell proliferation in mixed lymphocyte reaction (MLR). As shown in Figure 4E and H, monocyte-derived CD1a^+^ cells, obtained in the absence of DSCs (Figure 4A), induced strong T cell proliferation. On the other hand, cells cultured with DSCs that did not undergo DC differentiation (CD14^+^, CD1a^−^ phenotype, Figure 4C) completely failed to induce T cell responses (Figure 4G and H). In those cases in which DSC induce an incomplete inhibition of DC differentiation (mixed CD14^+^, CD1a^+^ phenotype, Figure 4B), only a partial T cell proliferation could be detected (Figure 4F and H). Altogether, these data indicate that DSC may exert an inhibitory effect on DC differentiation, thus suggesting their involvement in preventing full DC maturation, at least in the majority of cases analyzed.

### 6. DSCs may Express IDO, Jagged-1 and Produce PGE2

**Figure 5:**
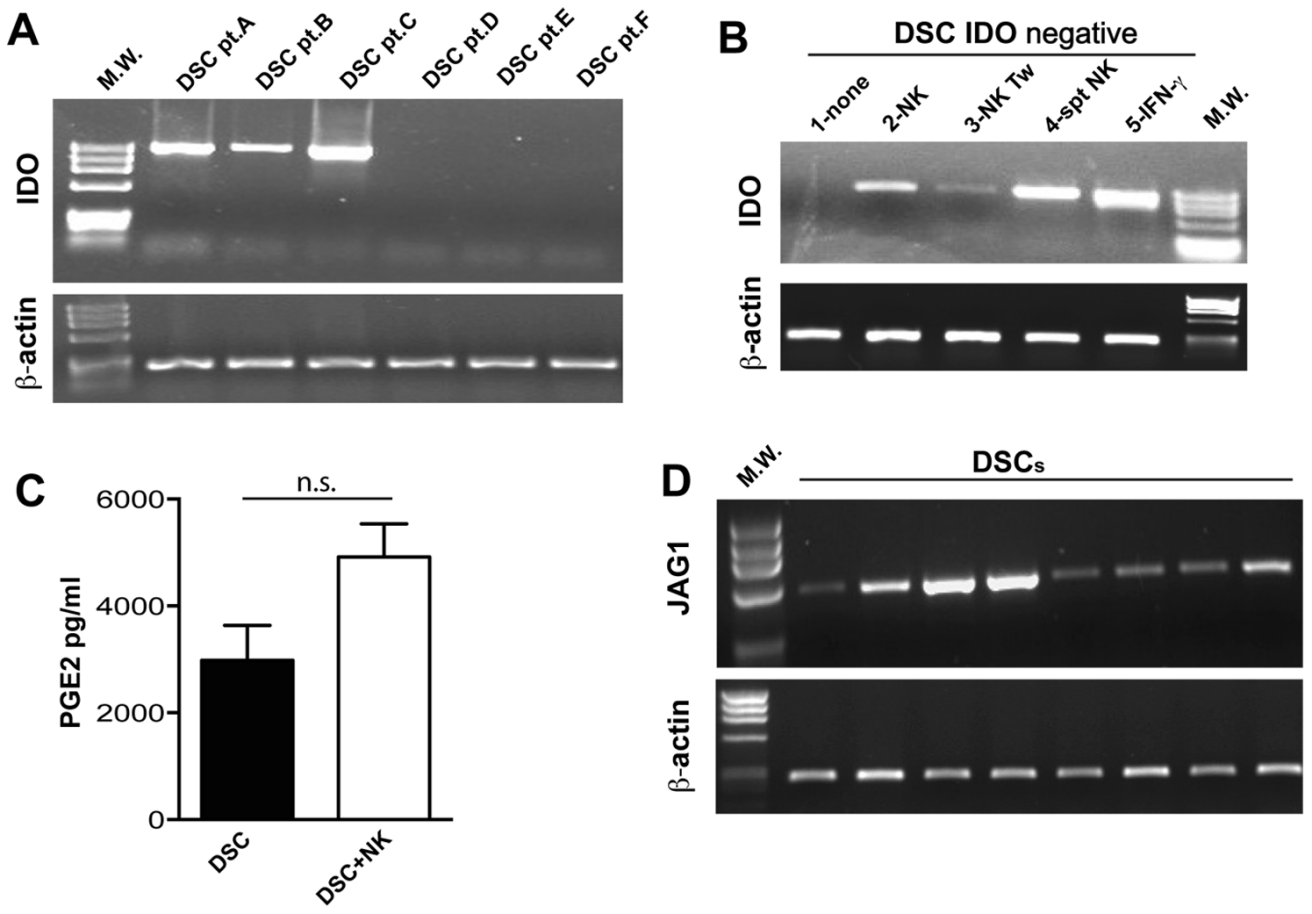
DSC express IDO and produce PGE2. (A) Analysis of IDO mRNA expression in DSCs isolated from different patients (pt). (B) IDO mRNA expression was analyzed in IDO^−^ DSCs cultured alone (first line) or with IL15-activated NK cells in cell-to-cell contact or Tw conditions or after ON incubation with supernatant (spt) of IL15-activated NK cells or 100 UI of IFN-γ. RT–PCR was performed with primers specific for IDO and for β-actin as positive control. PCR products were run on a 0.8% agarose gel and visualized by ethidium bromide staining. (C) PGE2 production was analyzed in supernatants by DSCs culture alone (black bar) or with IL15-activated NK cells. (D) Analysis of Jagged-1 mRNA expression in DSCs isolated from different patients.

In order to investigate the molecular mechanism(s) underlying the DSC-mediated inhibition of NK-cell function and DC differentiation, we analyzed whether inhibitory soluble factors or cellular interactions were involved in this phenomenon. Evidence exists that PGE2 and IDO-derived L-kynurenine represent important factors involved in the SC-mediated inhibitory effect on different cell types. We then analyzed the IDO mRNA expression in DSCs isolated from different patients (pt). Figure 5A shows that some, but not all, DSCs constitutively express IDO mRNA. Since IFN-γ is known to induce IDO expression, IDO^−^ DSCs were stimulated with different concentrations of this cytokine (from 1 to 100 IU/ml). After overnight incubation, IDO^−^ DSCs acquired IDO mRNA even at low IFN-γ concentrations (data not shown). As shown in figure 3D, PB-NK cells co-cultured with DSCs produced low amounts of IFN-γ. We evaluated whether these cytokine concentrations were sufficient to induce IDO expression. To this end, IDO^−^ DSCs were analyzed after co-culture with NK cells both under cell-to-cell contact and under transwell conditions. As shown in figure 5B, IDO mRNA was detectable under both conditions (lines 2 and 3). Similar results were obtained using supernatants of IL15-cultured PB-NK cells (line 4). We also investigated whether DSCs were able to produce PGE2. The large majority of DSCs were found to constitutively release PGE2. In addition, after co-culture with NK cells the amount of PGE2 was considerably higher than DSCs cultured alone (Figure 5C). Previous studies reported that SCs (in particular MSC from BM) could mediate their inhibitory function thanks to the expression of the Notch ligand, Jagged-1 [47], [56]. To this end, we analyzed by PCR the expression of Jagged-1 on DSC. As shown in figure 5D, Jagged-1 was expressed by all DSC analyzed (isolated from different donors).

### 7. Role of IDO, PGE2 and Jagged-1 in the Immunoregulatory Activity of DSCs

**Figure 6:**
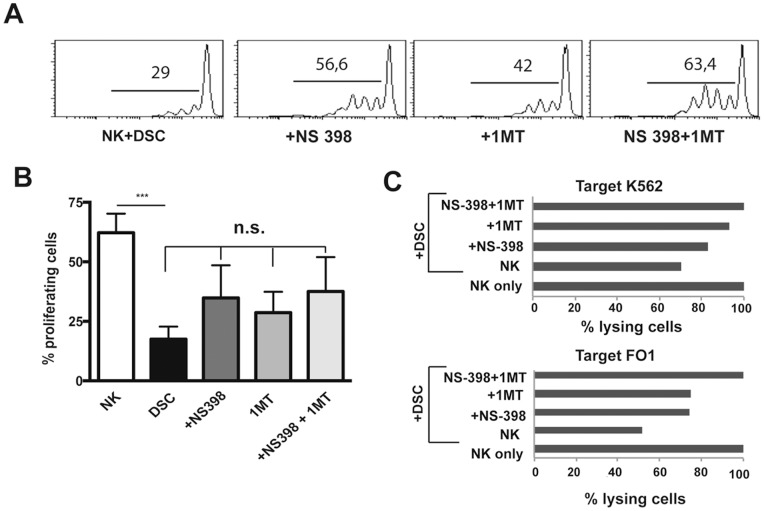
Role of IDO and PGE2 in the DSC-mediated inhibition of NK-cell functions. PB IL15-activated NK cells were cultured with DSCs in the presence of in the absence of IDO and PGE2 inhibitors. (A) After 7 days of cultured, proliferation of CFSE-labeled NK cells was analyzed. One representative experiment out of 3 performed. (B) Statistical analysis of proliferating CFSE-labeled NK cells ± SEM. (C) Cytolytic activity of IL15-activated NK cells cultured with DSC in the presence or in the absence of IDO and/or PGE2 inhibitors. ^51^Cr release against K562 and FO1 target cell lines was evaluated. One representative experiment out of 3 performed. Since we analyzed the inhibitory potential of different DSC, we considered, in each experiment, the cytotoxicity of IL15-activated NK cells cultured alone as 100%. The percentages of cytotoxicity of NK cells cultured in the presence of DSC ± inhibitors were normalized respect to IL15-activated NK cells cultured alone (set to 100%).

We next performed experiments aimed at investigating the actual role of IDO and/or PGE2 and/or Jagged-1 in DSC-mediated inhibition. To this hand, we used NS-398, an inhibitor of PGE2 synthesis, 1-methyl tryptophan (1-MT), an inhibitor of IDO enzymatic activity and Jagged-1 neutralizing mAb. PB-NK cells were cultured with IL15 in the presence or in the absence of DSCs. Inhibitors were added to the cultures either alone or in combination. However, the expression of the main activating NK receptors was not affected by the presence of the inhibitors (Figure S2 and S3A), NK cell proliferation was partially restored by 1-MT or NS-398 inhibitor and the effect was potentiated by the simultaneous blocking of IDO and PGE2 (Figure 6A–B). On the other hand, in the presence of Jagged-1 neutralizing mAb cell proliferation was not influenced (Figure S3B). We further investigated the effect of the inhibitors on the NK-mediated cytolytic activity. These experiments were performed using K562 and FO1 cell lines as targets. Partial restoration of cytolytic activity was detectable using NS-398 or 1-MT alone. The simultaneous blocking of IDO and PGE2 resulted in a complete restoration of cytolytic activity (Figure 6C). These results indicate that both IDO and PGE2 are involved in the DSC-mediated inhibition of NK-cell proliferation and cytotoxicity.

**Figure 7:**
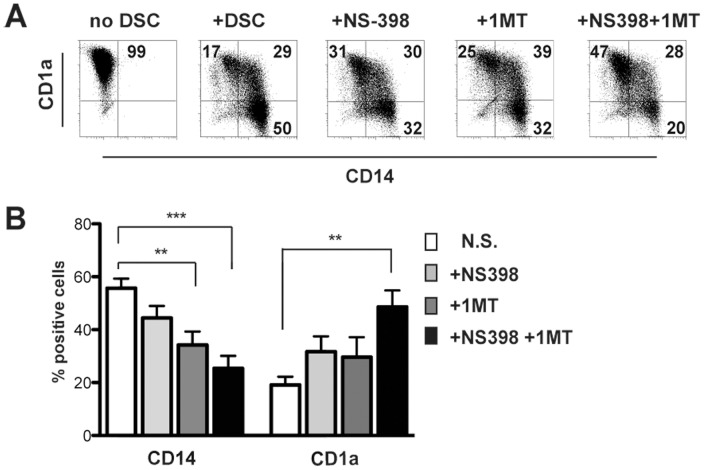
Role of IDO and PGE2 in the DC differentiation. PB-CD14^+^ cells were cultured with DSC, GM-CSF and IL4 for 5 days in the presence or in the absence of IDO and/or PGE2 inhibitors. (A) Analysis of CD1a and CD14 surface expression. One representative experiment out of 7 performed. (B) Statistical analysis of CD14 and CD1a markers. Data indicate the percentages of positive cells ± SEM of 7 independent experiments.

Finally, we analyzed the role of IDO, PGE2 and Jagged-1 in the DSC-mediated inhibition of DC differentiation. 1-MT and/or NS-398 were added to monocyte/DSC co-cultures. As shown in Figure 7A and B, cells displayed a high CD1a and low CD14 expression only when cultured with IDO and PGE2 inhibitors in combination. These data strongly suggest that PGE2 and IDO play an important role also in DSC-mediated inhibition of DC differentiation and function. On the contrary, the presence of anti-Jagged-1 neutralizing mAb did not affect DC differentiation (Figure S4).

## Discussion

In the present study we investigated whether DSCs may exert an effect on cells, such as NK and CD14^+^ myeloid cells, that are thought to play an important role in the induction of fetal tolerance during the first trimester of pregnancy [10], [20]. We show that DSCs inhibit the expression of major activating NK receptors and sharply suppress NK cell proliferation, cytotoxicity and cytokine production. In addition, DSCs block DC differentiation from monocytes, thus interfering with the acquisition of an important DC function i.e. their ability to elicit T cell responses.

Previous studies indicated that during the first trimester of pregnancy, decidual tissues are rich of particular cell types including NK cells and CD14^+^ myelomonocytic cells [10], [12], [20], [27]. Phenotypic and functional studies revealed that dNK cells display a peculiar phenotype and unusual functional features [10]–[14], [20]. Thus, although they express high amounts of CD56 antigen (CD56^bright^), at variance with peripheral blood CD56^bright^ NK cells, dNK are characterized by a high perforin and granzymes content and KIR^+^ expression [10], [13], [57]. Functional analysis showed that dNK cells are poorly cytolytic in spite of their high granule content [10]. This has been tentatively explained with the expression of an inhibitory form of 2B4 (CD244), the inability to form functional immunological synapses or the particular decidual microenvironment (hormones, cytokines and soluble factors) [4], [17], [18]. Perhaps more importantly, dNK cells release a peculiar set of cytokines/chemokines that play a major role in neoangiogenesis, tissue remodeling and placentation [13], [21]. On the other hand, they are poor producers of proinflammatory cytokines [10], [11], [13], [20]. Another important functional property of dNK cells is related to the immunosuppressive mechanisms involved in the maintenance of pregnancy. Thus, it has been shown that the cross-talk between dNK and dCD14^+^ cells results in the induction of potent inhibitory signals mediated by the dCD14^+^ cells themselves and by Tregs that are induced as a consequence of dNK/dCD14^+^ cell interactions [31]. Regarding the dCD14^+^ myelomonocytic cells, they display a particular surface phenotype, intermediate between M2 macrophages and DC [27]–[31]. Importantly, they display IDO activity (resulting in the release of L-kynurenine) and produce TGF-β as a consequence of their cross-talk with dNK cells [31].

SCs represent another cell type abundantly present in decidual tissues. We have recently shown that these DSCs can sustain differentiation of CD34^+^ cell precursors isolated from decidua towards NK cells [22]. Since the particular phenotypic and functional characteristics of dNK and dCD14^+^ cells may be consequent to the effect of the decidual microenvironment, we speculated that DSC could play an important role in such microenvironment. Indeed, our present study provides clear evidence that DSCs exert a marked regulatory role on both peripheral NK and CD14^+^ cells. In particular, we show that upon interaction with DSC, NK cells display: 1) down-regulation of major activating receptors, 2) an impaired proliferating capability, 3) a compromised cytolytic activity 4) a reduced IFN-γ and increased IL-8 production. Since NK cells used in the present experimental setting were obtained from PB of unrelated healthy donors, our results indicate that DSC can exert their modulatory activity also on mature NK cells. This information is important because it is still unclear whether dNK cells derive only from decidual CD34^+^ precursors or also from PB-NK cells migrating to decidua when pregnancy is established [20], [22]–[25]. The two hypotheses may be true, indeed our present data indicate that DSCs (and the decidual microenvironment) may inhibit cytotoxicity and pro-inflammatory cytokine production in peripheral blood NK cells potentially capable of migrating to decidua when pregnancy establishes, thus preventing possible NK-mediated damages. In addition upon interaction with DSCs, these NK cells may acquire functional properties typical of dNK cells and a may play an important role in the invasion, proliferation and differentiation of trophoblast cells.

Regarding the ability of DSC to block DC differentiation from peripheral monocytes, this function is particularly relevant because the presence of mature DC in placental tissues may induce potentially harmful T cell responses, leading to sensitization to maternal T cells to placental or paternal antigens.

The immunomodulatory effects of SC could involve both cell contact and soluble factors, including IDO, TGF-β, IL-10, PGE2, NO, Jagged-1 and chemokines/citokines [26], [39]–[41], [45]–[47]. These soluble factors could mediate the inhibitory effect exerted by DSCs on different cells of the innate or adaptive immune system. In this contest, we analyzed the production of PGE2 by DSCs and observed that DSCs produce PGE2 constitutively and that production increases when DSCs are co-cultured with NK cells. In addition, the majority but not all DSCs constitutively expressed IDO but after IFN-γstimulation (produced by NK cells), IDO^−^ DSC acquired IDO expression. Therefore, we analyze the possible involvement of IDO and PGE2 in DSC/NK or DSC/CD14^+^ co-cultures. Our results indicate that both IDO and PGE2 would play an important role in the inhibition of NK-cell proliferation and function and in DC differentiation. Indeed, in the presence of the PGE2 inhibitor NS-398 and IDO inhibitors 1-MT (added to DSC/NK or DSC/CD14^+^ co-cultures), a complete restoration of NK cell functions and DC differentiation could be detected.

Our results suggest that DSCs, may contribute to the induction of an anti-inflammatory and tolerogenic environment during early pregnancy. Moreover, based on our present results, it is possible to speculate that impairment of DSCs function may result in miscarriages due to immunological mechanisms. Finally, in view of the potent immunomodulatory activity of DSC on DC maturation, it is conceivable that they may represent a suitable source of SC for cell-based treatment of steroid resistant GVHD.

## Supporting Information

Figure S1 **IL15 receptors and cytokines expression by PB-NK cells.** (A) Expression of IL15R on PB-NK cells cultured in the absence (white profiles) or in the presence of DSCs (grey profiles) for 24 or 48 hours. One representative experiment out of 3 performed. (B-C) IFN-γ production, after 4 h of co-culture with K562 and FO1 target cell lines by IL15-activated NK cells cultured in the absence (white bars) or in the presence (black bars) of DSCs. (D) IL-8 positive cells, after 4 h of culture with PMA/ionomycin in IL15-activated NK cells cultured in the absence (white bars) or in the presence (black bars) of DSCs. (E) Real-time (RT)-PCR analysis of VEGF in IL15-activated NK cells cultured in the absence (white bars) or in the presence (black bars) of DSCs. For each group of cells, we calculated the sample relative expression on the basis of the expression level detected in NK cells cultured alone, arbitrarily normalized to 1. Data were obtained from 4 independent experiments.
(TIF)

Figure S2 **Role of IDO and PGE2 in the DSC-mediated inhibition of NK cell activating receptors.** Expression of NKp46, NKp30, NKp44, NKG2D and DNAM-1 on IL15-activated PB-NK cells, at day 5 of culture, in the absence (white profiles) or in the presence of DSCs (grey profiles) with IDO and/or PGE2 inhibitor. Cells were analyzed by gating on CD56^+^CD3^−^ cells. One representative experiment out of 9 performed.
(TIF)

Figure S3 **Role of Jagged-1 in the DSC-mediated inhibition of NK cell activating receptors.** IL15-activated PB-NK cells were cultured with DSCs in the presence of in the absence of Jagged-1 neutralizing mAb. (A) Expression of NKp46, NKp30, NKp44, NKG2D and DNAM-1 on IL15-activated PB-NK cells, at day 5 of culture, in the absence (white profiles) or in the presence of DSCs (grey profiles) ± Jagged-1 neutralizing mAb. Cells were analyzed by gating on CD56^+^CD3^−^ cells. One representative experiment out of 4 performed. (B) After 7 days of culture, proliferation of CFSE-labeled PB-NK cells was analyzed. One representative experiment out of 4 performed.
(TIF)

Figure S4 **Role of Jagged-1 in the DC differentiation.** PB-CD14^+^ cells were cultured with DSC, GM-CSF and IL4 for 5 days in the presence or in the absence of Jagged-1 neutralizing mAb. Statistical analysis of CD14 and CD1a markers. Data indicate the percentages of positive cells ± SEM of 4 independent experiments.
(TIF)

## References

[1] Caligiuri MA (2008) Human natural killer cells. Blood 112: 461–469. 18650461 10.1182/blood-2007-09-077438 PMC2481557

[2] Cerwenka A, Lanier LL (2001) Natural killer cells, viruses and cancer. Nat Rev Immunol 1: 41–49. 11905813 10.1038/35095564

[3] Fauriat C, Long EO, Ljunggren HG, Bryceson YT (2010) Regulation of human NK-cell cytokine and chemokine production by target cell recognition. Blood 115: 2167–2176. 19965656 10.1182/blood-2009-08-238469 PMC2844017

[4] Le Bouteiller P, Tabiasco J (2006) Killers become builders during pregnancy. Nat Med 12: 991–992. 16960566 10.1038/nm0906-991

[5] Moretta A (2002) Natural killer cells and dendritic cells: rendezvous in abused tissues. Nat Rev Immunol 2: 957–964. 12461568 10.1038/nri956

[6] Moretta A, Bottino C, Vitale M, Pende D, Biassoni R, et al (1996) Receptors for HLA class-I molecules in human natural killer cells. Annu Rev Immunol 14: 619–648. 8717527 10.1146/annurev.immunol.14.1.619

[7] Moretta A, Bottino C, Vitale M, Pende D, Cantoni C, et al (2001) Activating receptors and coreceptors involved in human natural killer cell-mediated cytolysis. Annu Rev Immunol 19: 197–223. 11244035 10.1146/annurev.immunol.19.1.197

[8] Moretta L, Moretta A (2004) Unravelling natural killer cell function: triggering and inhibitory human NK receptors. EMBO J 23: 255–259. 14685277 10.1038/sj.emboj.7600019 PMC1271745

[9] Lanier LL (2005) NK cell recognition. Annu Rev Immunol 23: 225–274. 15771571 10.1146/annurev.immunol.23.021704.115526

[10] Moffett-King A (2002) Natural killer cells and pregnancy. Nat Rev Immunol 2: 656–663. 12209134 10.1038/nri886

[11] Hanna J, Goldman-Wohl D, Hamani Y, Avraham I, Greenfield C, et al (2006) Decidual NK cells regulate key developmental processes at the human fetal-maternal interface. Nat Med 12: 1065–1074. 16892062 10.1038/nm1452

[12] Bulmer JN, Williams PJ, Lash GE (2010) Immune cells in the placental bed. Int J Dev Biol 54: 281–294. 19876837 10.1387/ijdb.082763jb

[13] Hanna J, Mandelboim O (2007) When killers become helpers. Trends Immunol 28: 201–206. 17403615 10.1016/j.it.2007.03.005

[14] Manaster I, Mandelboim O (2010) The unique properties of uterine NK cells. Am J Reprod Immunol 63: 434–444. 20055791 10.1111/j.1600-0897.2009.00794.x

[15] Vacca P, Cantoni C, Prato C, Fulcheri E, Moretta A, et al (2008) Regulatory role of NKp44, NKp46, DNAM-1 and NKG2D receptors in the interaction between NK cells and trophoblast cells. Evidence for divergent functional profiles of decidual versus peripheral NK cells. Int Immunol 20: 1395–1405. 18815119 10.1093/intimm/dxn105

[16] Koopman LA, Kopcow HD, Rybalov B, Boyson JE, Orange JS, et al (2003) Human decidual natural killer cells are a unique NK cell subset with immunomodulatory potential. J Exp Med 198: 1201–1212. 14568979 10.1084/jem.20030305 PMC2194228

[17] Vacca P, Pietra G, Falco M, Romeo E, Bottino C, et al (2006) Analysis of natural killer cells isolated from human decidua: Evidence that 2B4 (CD244) functions as an inhibitory receptor and blocks NK-cell function. Blood 108: 4078–4085. 16931625 10.1182/blood-2006-04-017343

[18] Kopcow HD, Allan DS, Chen X, Rybalov B, Andzelm MM, et al (2005) Human decidual NK cells form immature activating synapses and are not cytotoxic. Proc Natl Acad Sci U S A 102: 15563–15568. 16230631 10.1073/pnas.0507835102 PMC1266146

[19] Moffett A, Loke C (2006) Immunology of placentation in eutherian mammals. Nat Rev Immunol 6: 584–594. 16868549 10.1038/nri1897

[20] Vacca P, Moretta L, Moretta A, Mingari MC (2011) Origin, phenotype and function of human natural killer cells in pregnancy. Trends Immunol 32: 517–523. 21889405 10.1016/j.it.2011.06.013

[21] Robson A, Harris LK, Innes BA, Lash GE, Aljunaidy MM, et al (2012) Uterine natural killer cells initiate spiral artery remodeling in human pregnancy. FASEB J 26: 4876–4885. 22919072 10.1096/fj.12-210310

[22] Vacca P, Vitale C, Montaldo E, Conte R, Cantoni C, et al (2011) CD34+ hematopoietic precursors are present in human decidua and differentiate into natural killer cells upon interaction with stromal cells. Proc Natl Acad Sci U S A 108: 2402–2407. 21248224 10.1073/pnas.1016257108 PMC3038730

[23] Manaster I, Mizrahi S, Goldman-Wohl D, Sela HY, Stern-Ginossar N, et al (2008) Endometrial NK cells are special immature cells that await pregnancy. J Immunol 181: 1869–1876. 18641324 10.4049/jimmunol.181.3.1869

[24] Carlino C, Stabile H, Morrone S, Bulla R, Soriani A, et al (2008) Recruitment of circulating NK cells through decidual tissues: a possible mechanism controlling NK cell accumulation in the uterus during early pregnancy. Blood 111: 3108–3115. 18187664 10.1182/blood-2007-08-105965

[25] Male V, Hughes T, McClory S, Colucci F, Caligiuri MA, et al (2010) Immature NK cells, capable of producing IL-22, are present in human uterine mucosa. J Immunol 185: 3913–3918. 20802153 10.4049/jimmunol.1001637 PMC3795409

[26] Keskin DB, Allan DS, Rybalov B, Andzelm MM, Stern JN, et al (2007) TGFbeta promotes conversion of CD16+ peripheral blood NK cells into CD16- NK cells with similarities to decidual NK cells. Proc Natl Acad Sci U S A 104: 3378–3383. 17360654 10.1073/pnas.0611098104 PMC1805591

[27] Kammerer U, Eggert AO, Kapp M, McLellan AD, Geijtenbeek TB, et al (2003) Unique appearance of proliferating antigen-presenting cells expressing DC-SIGN (CD209) in the decidua of early human pregnancy. Am J Pathol 162: 887–896. 12598322 10.1016/S0002-9440(10)63884-9 PMC1868095

[28] Laskarin G, Cupurdija K, Tokmadzic VS, Dorcic D, Dupor J, et al (2005) The presence of functional mannose receptor on macrophages at the maternal-fetal interface. Hum Reprod 20: 1057–1066. 15746201 10.1093/humrep/deh740

[29] Gustafsson C, Mjosberg J, Matussek A, Geffers R, Matthiesen L, et al (2008) Gene expression profiling of human decidual macrophages: evidence for immunosuppressive phenotype. PLoS One 3: e2078. 18446208 10.1371/journal.pone.0002078 PMC2323105

[30] Laskarin G, Redzovic A, Rubesa Z, Mantovani A, Allavena P, et al (2008) Decidual natural killer cell tuning by autologous dendritic cells. Am J Reprod Immunol 59: 433–445. 18405314 10.1111/j.1600-0897.2008.00599.x

[31] Vacca P, Cantoni C, Vitale M, Prato C, Canegallo F, et al (2010) Crosstalk between decidual NK and CD14+ myelomonocytic cells results in induction of Tregs and immunosuppression. Proc Natl Acad Sci U S A 107: 11918–11923. 20547831 10.1073/pnas.1001749107 PMC2900704

[32] Saito S, Sakai M, Sasaki Y, Nakashima A, Shiozaki A (2007) Inadequate tolerance induction may induce pre-eclampsia. J Reprod Immunol 76: 30–39. 17935792 10.1016/j.jri.2007.08.002

[33] Heikkinen J, Mottonen M, Alanen A, Lassila O (2004) Phenotypic characterization of regulatory T cells in the human decidua. Clin Exp Immunol 136: 373–378. 15086404 10.1111/j.1365-2249.2004.02441.x PMC1809024

[34] Erkers T, Nava S, Yosef J, Ringden O, Kaipe H (2013) Decidual stromal cells promote regulatory T cells and suppress alloreactivity in a cell contact-dependent manner. Stem Cells Dev 22: 2596–2605. 23701127 10.1089/scd.2013.0079

[35] Gronthos S, Franklin DM, Leddy HA, Robey PG, Storms RW, et al (2001) Surface protein characterization of human adipose tissue-derived stromal cells. J Cell Physiol 189: 54–63. 11573204 10.1002/jcp.1138

[36] Pittenger MF, Mackay AM, Beck SC, Jaiswal RK, Douglas R, et al (1999) Multilineage potential of adult human mesenchymal stem cells. Science 284: 143–147. 10102814 10.1126/science.284.5411.143

[37] Deans RJ, Moseley AB (2000) Mesenchymal stem cells: biology and potential clinical uses. Exp Hematol 28: 875–884. 10989188 10.1016/s0301-472x(00)00482-3

[38] Uccelli A, Moretta L, Pistoia V (2006) Immunoregulatory function of mesenchymal stem cells. Eur J Immunol 36: 2566–2573. 17013987 10.1002/eji.200636416

[39] Xu X, Wang Q, Deng B, Wang H, Dong Z, et al (2012) Monocyte chemoattractant protein-1 secreted by decidual stromal cells inhibits NK cells cytotoxicity by up-regulating expression of SOCS3. PLoS One 7: e41869. 22848642 10.1371/journal.pone.0041869 PMC3407114

[40] Olivares EG, Montes MJ, Oliver C, Galindo JA, Ruiz C (1997) Cultured human decidual stromal cells express B7–1 (CD80) and B7–2 (CD86) and stimulate allogeneic T cells. Biol Reprod 57: 609–615. 9282998 10.1095/biolreprod57.3.609

[41] Balsamo M, Scordamaglia F, Pietra G, Manzini C, Cantoni C, et al (2009) Melanoma-associated fibroblasts modulate NK cell phenotype and antitumor cytotoxicity. Proc Natl Acad Sci U S A 106: 20847–20852. 19934056 10.1073/pnas.0906481106 PMC2791633

[42] Dokic J, Tomic S, Markovic M, Milosavljevic P, Colic M (2013) Mesenchymal stem cells from periapical lesions modulate differentiation and functional properties of monocyte-derived dendritic cells. Eur J Immunol 43: 1862–1872. 23616249 10.1002/eji.201243010

[43] Erices A, Conget P, Minguell JJ (2000) Mesenchymal progenitor cells in human umbilical cord blood. Br J Haematol 109: 235–242. 10848804 10.1046/j.1365-2141.2000.01986.x

[44] Di Nicola M, Carlo-Stella C, Magni M, Milanesi M, Longoni PD, et al (2002) Human bone marrow stromal cells suppress T-lymphocyte proliferation induced by cellular or nonspecific mitogenic stimuli. Blood 99: 3838–3843. 11986244 10.1182/blood.v99.10.3838

[45] Spaggiari GM, Capobianco A, Abdelrazik H, Becchetti F, Mingari MC, et al (2008) Mesenchymal stem cells inhibit natural killer-cell proliferation, cytotoxicity, and cytokine production: role of indoleamine 2,3-dioxygenase and prostaglandin E2. Blood 111: 1327–1333. 17951526 10.1182/blood-2007-02-074997

[46] Spaggiari GM, Abdelrazik H, Becchetti F, Moretta L (2009) MSCs inhibit monocyte-derived DC maturation and function by selectively interfering with the generation of immature DCs: central role of MSC-derived prostaglandin E2. Blood 113: 6576–6583. 19398717 10.1182/blood-2009-02-203943

[47] Liotta F, Angeli R, Cosmi L, Fili L, Manuelli C, et al (2008) Toll-like receptors 3 and 4 are expressed by human bone marrow-derived mesenchymal stem cells and can inhibit their T-cell modulatory activity by impairing Notch signaling. Stem Cells 26: 279–289. 17962701 10.1634/stemcells.2007-0454

[48] Amsen D, Blander JM, Lee GR, Tanigaki K, Honjo T, et al (2004) Instruction of distinct CD4 T helper cell fates by different notch ligands on antigen-presenting cells. Cell 117: 515–526. 15137944 10.1016/s0092-8674(04)00451-9

[49] Manaster I, Gazit R, Goldman-Wohl D, Stern-Ginossar N, Mizrahi S, et al (2010) Notch activation enhances IFNgamma secretion by human peripheral blood and decidual NK cells. J Reprod Immunol 84: 1–7. 20004979 10.1016/j.jri.2009.10.009

[50] He YY, He XJ, Guo PF, Du MR, Shao J, et al (2012) The decidual stromal cells-secreted CCL2 induces and maintains decidual leukocytes into Th2 bias in human early pregnancy. Clin Immunol 145: 161–173. 23069648 10.1016/j.clim.2012.07.017

[51] Kayisli UA, Selam B, Guzeloglu-Kayisli O, Demir R, Arici A (2003) Human chorionic gonadotropin contributes to maternal immunotolerance and endometrial apoptosis by regulating Fas-Fas ligand system. J Immunol 171: 2305–2313. 12928375 10.4049/jimmunol.171.5.2305

[52] D’Urso CM, Wang ZG, Cao Y, Tatake R, Zeff RA, et al (1991) Lack of HLA class I antigen expression by cultured melanoma cells FO-1 due to a defect in B2m gene expression. J Clin Invest 87: 284–292. 1898655 10.1172/JCI114984 PMC295046

[53] Wang Z, Hu XZ, Tatake RJ, Yang SY, Zeff RA, et al (1993) Differential effect of human and mouse beta 2-microglobulin on the induction and the antigenic profile of endogenous HLA-A and -B antigens synthesized by beta 2-microglobulin gene-null FO-1 melanoma cells. Cancer Res 53: 4303–4309. 7689931

[54] Can A, Tekelioglu M, Baltaci A (1995) Expression of desmin and vimentin intermediate filaments in human decidual cells during first trimester pregnancy. Placenta 16: 261–275. 7638108 10.1016/0143-4004(95)90113-2

[55] Oliver C, Montes MJ, Galindo JA, Ruiz C, Olivares EG (1999) Human decidual stromal cells express alpha-smooth muscle actin and show ultrastructural similarities with myofibroblasts. Hum Reprod 14: 1599–1605. 10357983 10.1093/humrep/14.6.1599

[56] Rutz S, Mordmuller B, Sakano S, Scheffold A (2005) Notch ligands Delta-like1, Delta-like4 and Jagged1 differentially regulate activation of peripheral T helper cells. Eur J Immunol 35: 2443–2451. 16047342 10.1002/eji.200526294

[57] Vacca P, Mingari MC, Moretta L (2013) Natural killer cells in human pregnancy. J Reprod Immunol 97: 14–19. 23432867 10.1016/j.jri.2012.10.008

