# Supplementary figures and images for: Stromal Cells from Human Decidua Exert a Strong Inhibitory Effect on NK Cell Function and Dendritic Cell Differentiation
Source: PLoS One. 2014 Feb 20;9(2):e89006. doi: 10.1371/journal.pone.0089006 (PMC3930605; doi:10.1371/journal.pone.0089006)

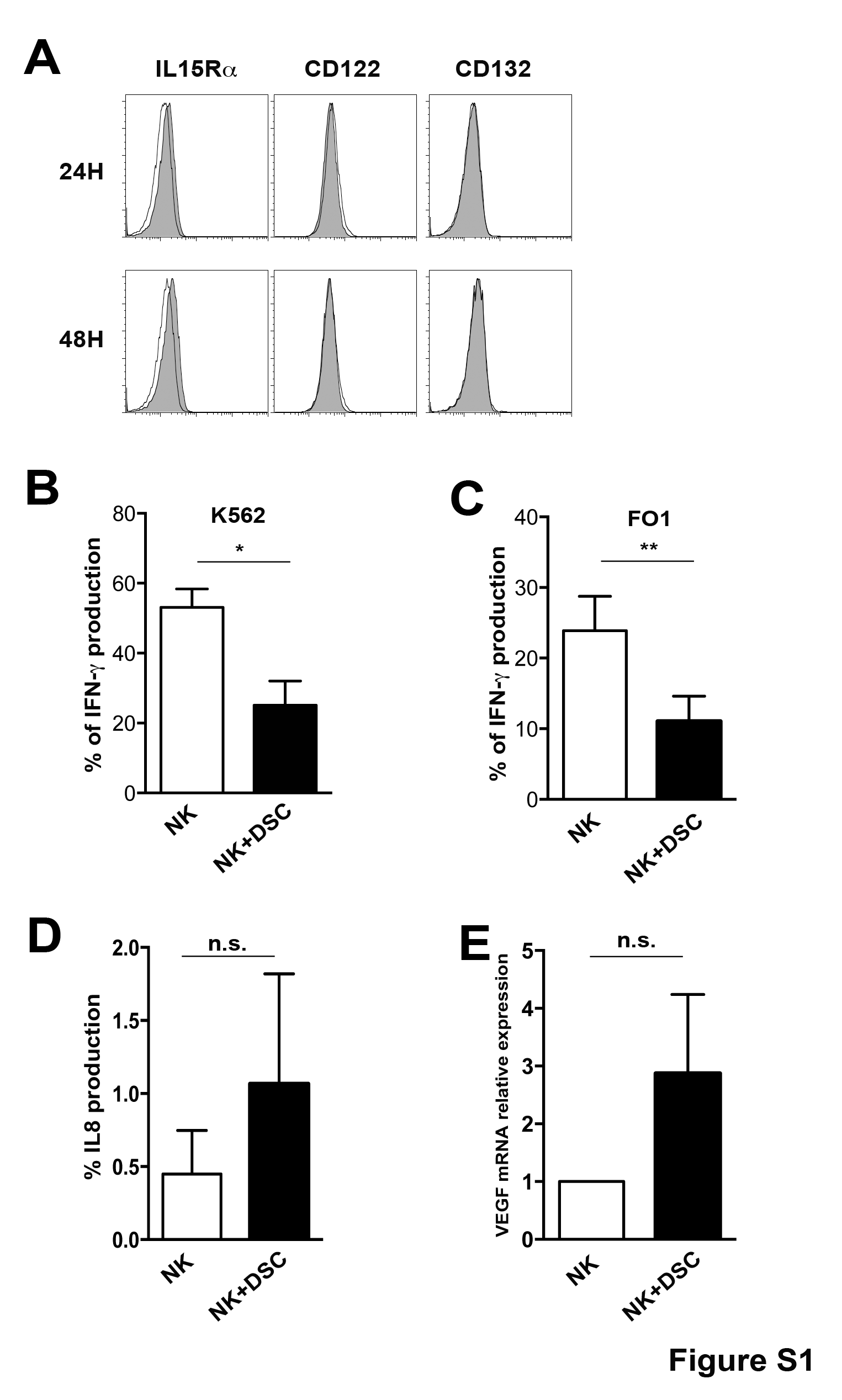

Supplement: Figure S1 — IL15 receptors and cytokines expression by PB-NK cells. (A) Expression of IL15R on PB-NK cells cultured in the absence (white profiles) or in the presence of DSCs (grey profiles) for 24 or 48 hours. One representative experiment out of 3 performed. (B-C) IFN-γ production, after 4 h of co-culture with K562 and FO1 target cell lines by IL15-activated NK cells cultured in the absence (white bars) or in the presence (black bars) of DSCs. (D) IL-8 positive cells, after 4 h of culture with PMA/ionomycin in IL15-activated NK cells cultured in the absence (white bars) or in the presence (black bars) of DSCs. (E) Real-time (RT)-PCR analysis of VEGF in IL15-activated NK cells cultured in the absence (white bars) or in the presence (black bars) of DSCs. For each group of cells, we calculated the sample relative expression on the basis of the expression level detected in NK cells cultured alone, arbitrarily normalized to 1. Data were obtained from 4 independent experiments. (TIF) [file pone.0089006.s001.tif]

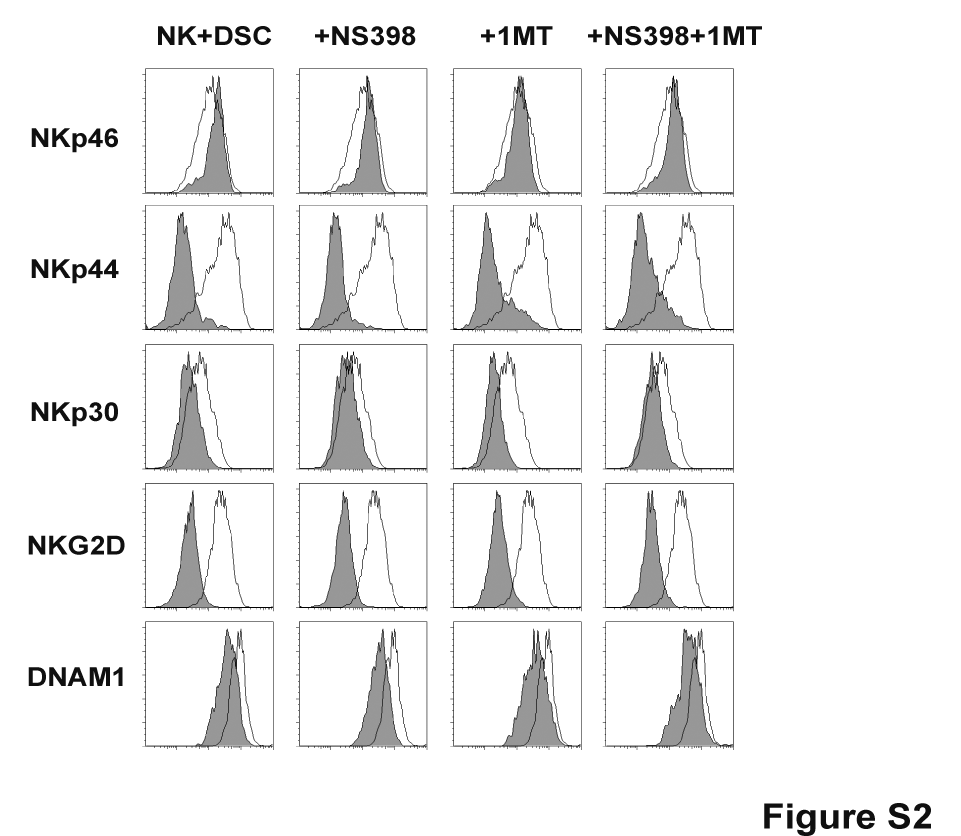

Supplement: Figure S2 — Role of IDO and PGE2 in the DSC-mediated inhibition of NK cell activating receptors. Expression of NKp46, NKp30, NKp44, NKG2D and DNAM-1 on IL15-activated PB-NK cells, at day 5 of culture, in the absence (white profiles) or in the presence of DSCs (grey profiles) with IDO and/or PGE2 inhibitor. Cells were analyzed by gating on CD56+CD3− cells. One representative experiment out of 9 performed. (TIF) [file pone.0089006.s002.tif]

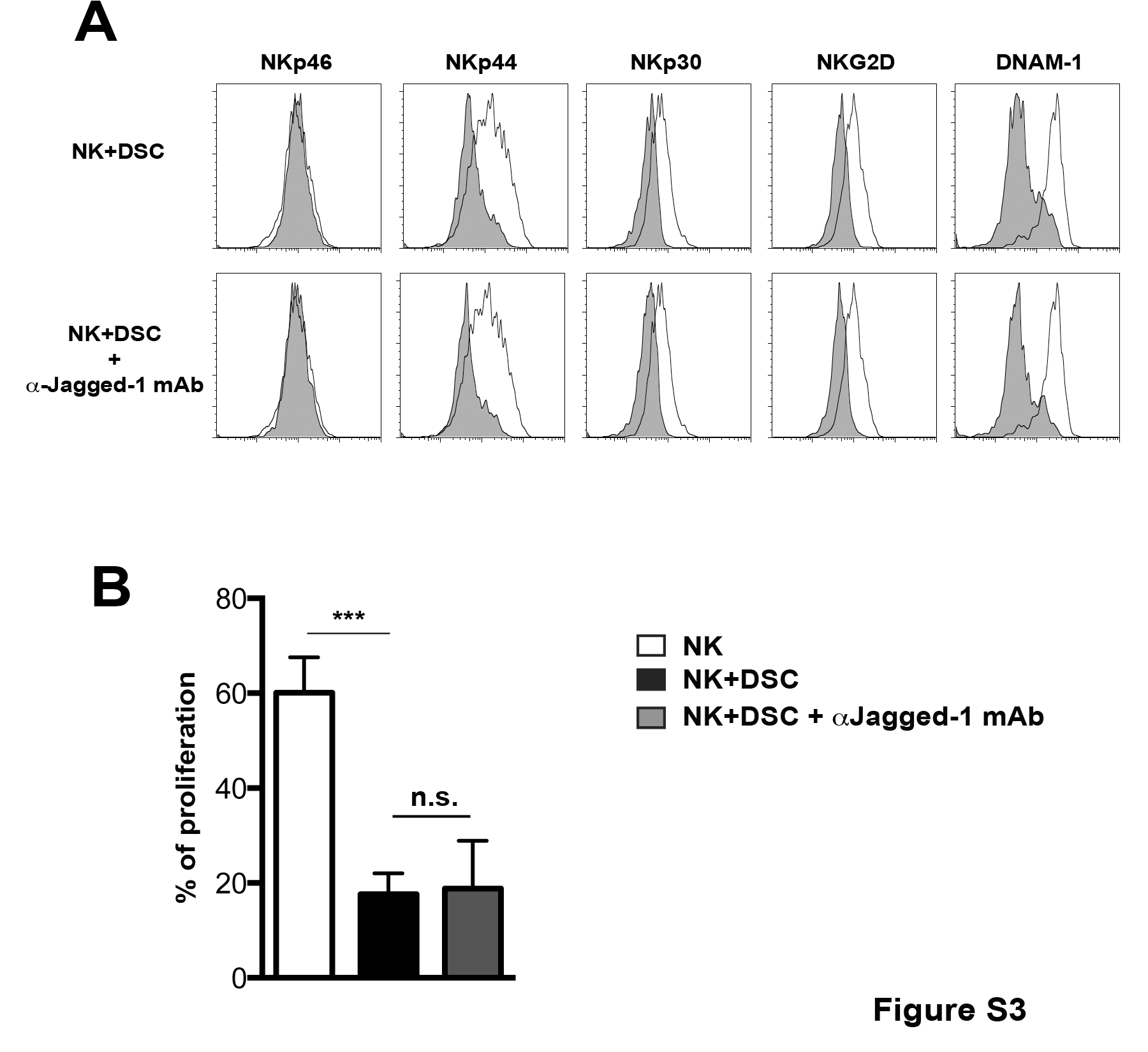

Supplement: Figure S3 — Role of Jagged-1 in the DSC-mediated inhibition of NK cell activating receptors. IL15-activated PB-NK cells were cultured with DSCs in the presence of in the absence of Jagged-1 neutralizing mAb. (A) Expression of NKp46, NKp30, NKp44, NKG2D and DNAM-1 on IL15-activated PB-NK cells, at day 5 of culture, in the absence (white profiles) or in the presence of DSCs (grey profiles) ± Jagged-1 neutralizing mAb. Cells were analyzed by gating on CD56+CD3− cells. One representative experiment out of 4 performed. (B) After 7 days of culture, proliferation of CFSE-labeled PB-NK cells was analyzed. One representative experiment out of 4 performed. (TIF) [file pone.0089006.s003.tif]

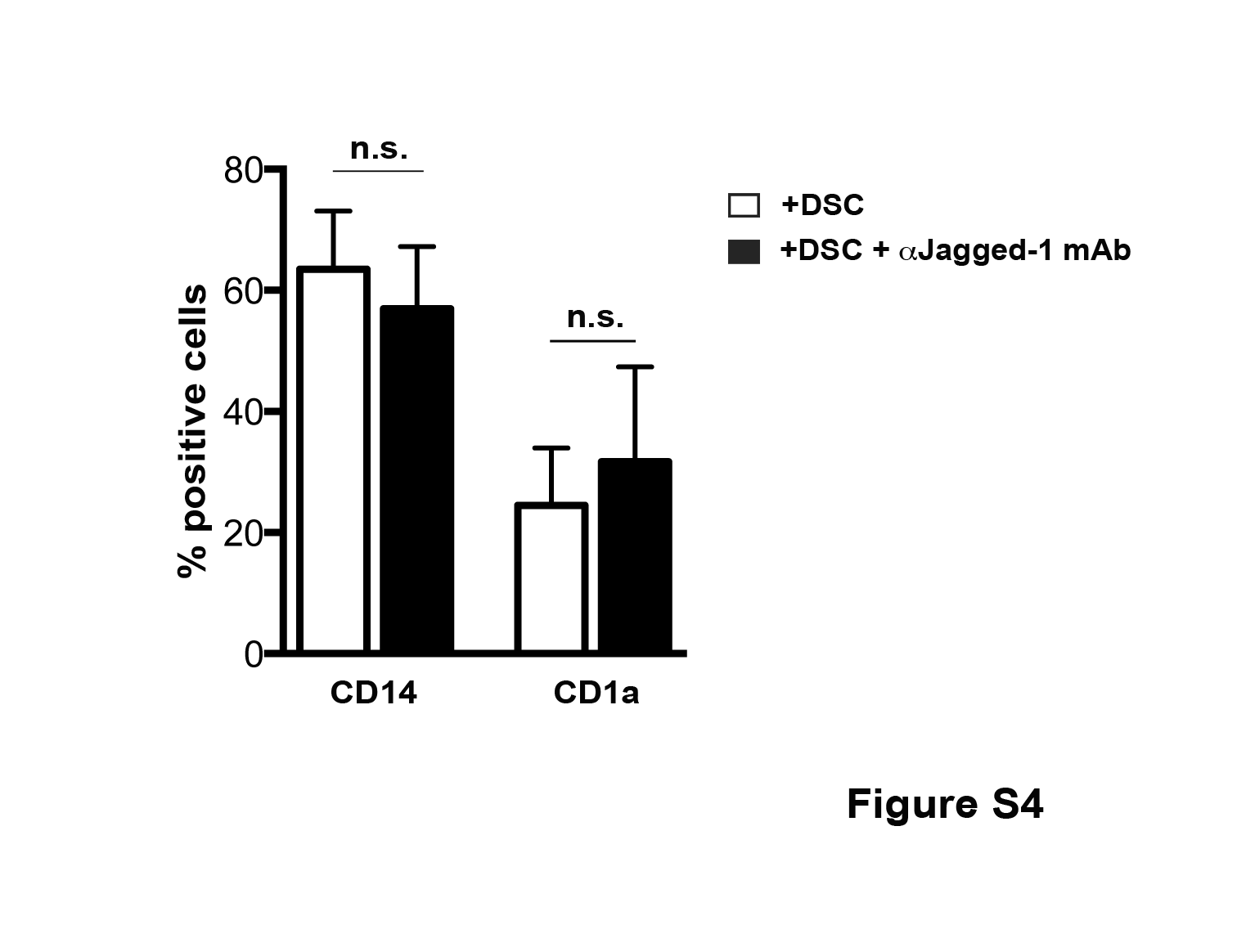

Supplement: Figure S4 — Role of Jagged-1 in the DC differentiation. PB-CD14+ cells were cultured with DSC, GM-CSF and IL4 for 5 days in the presence or in the absence of Jagged-1 neutralizing mAb. Statistical analysis of CD14 and CD1a markers. Data indicate the percentages of positive cells ± SEM of 4 independent experiments. (TIF) [file pone.0089006.s004.tif]
